# P-37 - SOCIODEMOGRAPHIC FACTORS ASSOCIATED WITH MPOX VACCINE UPTAKE AMONG GAY AND BISEXUAL MEN WHO HAVE SEX WITH MEN (GBMSM) IN SCOTLAND

**DOI:** 10.1093/pubmed/fdag046.083

**Published:** 2026-07-09

**Authors:** Miss Vera Chua, Dr Kirsty Morrison, Dr Taimoor Hasan, Mr James Munro, Miss Michelle Falconer, Dr Claire Cameron, Dr Cheryl Gibbons

**Affiliations:** Public Health Scotland; Public Health Scotland; Public Health Scotland; Public Health Scotland; Public Health Scotland; Public Health Scotland; Public Health Scotland

## Abstract

**Background:**

Mpox has spread globally since 2022. Outside sub-Saharan Africa, mpox has primarily affected gay, bisexual, and other men who have sex with men (GBMSM) communities. Scotland introduced a targeted vaccination programme in June 2022 to provide protection against mpox within the high-risk GBMSM community.

**Objectives:**

We assessed two-dose mpox vaccine uptake among high-risk GBMSM and identified sociodemographic factors associated with vaccine uptake in Scotland.

**Methods:**

We conducted a retrospective cross-sectional study of a cohort of high-risk eligible GBMSM. Multivariable logistic regression estimated adjusted odds ratios (aORs) for being unvaccinated and for being fully vaccinated (two doses) between July 2022 and December 2023.

**Results:**

Among 8,846 eligible GBMSM, 4,846 were unvaccinated. Of 4,000 who received ≥1 dose, 1,743 received one dose and 2,257 completed two doses.

Higher odds of being unvaccinated were associated with age ≤24 years (reference: aged 55+ years, aOR: 3.51, 95% CI: 2.89-4.25), living in the most deprived quintile (reference: least deprived; aOR 1.46, 95% CI 1.26–1.71), eligibility via recent bacterial STI (reference: PrEP; aOR 2.98, 95% CI 2.62–3.40), and reporting sexual partners of both men and women (reference: men only; aOR 1.63, 95% CI 1.46–1.82).

Lower odds of completing two doses after receiving one were associated with living in the most deprived quintile (aOR 0.58, 95% CI 0.47–0.72), eligibility via recent bacterial STI (PrEP; aOR 0.67, 95% CI 0.53–0.83), and reporting sexual partners of both men and women (aOR 0.75, 95% CI 0.63–0.88).

**Conclusions:**

Socioeconomic deprivation, younger age, eligibility pathway, and bisexual partner history were associated with non-uptake and/or partial uptake. These findings can help target interventions to improve uptake and dose completion; further work should explore reasons behind vaccine uptake decisions.

